# Children and young people who die by suicide: childhood-related antecedents, gender differences and service contact

**DOI:** 10.1192/bjo.2020.33

**Published:** 2020-05-11

**Authors:** Cathryn Rodway, Su-Gwan Tham, Saied Ibrahim, Pauline Turnbull, Nav Kapur, Louis Appleby

**Affiliations:** National Confidential Inquiry into Suicide and Safety in Mental Health (NCISH), Centre for Mental Health and Safety, School of Health Sciences, University of Manchester, UK; National Confidential Inquiry into Suicide and Safety in Mental Health (NCISH), Centre for Mental Health and Safety, School of Health Sciences, University of Manchester; and Greater Manchester Mental Health NHS Foundation Trust, UK

**Keywords:** Suicide, childhood experience, deliberate self-harm, epidemiology, trauma

## Abstract

**Background:**

Worldwide suicide is commonest in young people and in many countries, including the UK, suicide rates in young people are rising.

**Aims:**

To investigate the stresses young people face before they take their lives, their contact with services that could be preventative and whether these differ in girls and boys.

**Method:**

We identified a 3-year UK national consecutive case series of deaths by suicide in people aged 10–19, based on national mortality data. We extracted information on the antecedents of suicide from official investigations, primarily inquests.

**Results:**

Between 2014 and 2016, there were 595 suicides by young people, almost 200 per year; 71% were male (*n* = 425). Suicide rates increased from the mid-teens, most deaths occurred in those aged 17–19 (443, 74%). We obtained data about the antecedents of suicide for 544 (91%). A number of previous and recent stresses were reported including witnessing domestic violence, bullying, self-harm, bereavement (including by suicide) and academic pressures. These experiences were generally more common in girls than boys, whereas drug misuse (odds ratio (OR) = 0.54, 95% CI 0.35–0.83, *P* = 0.006) and workplace problems (OR 0.52, 95% CI 0.28–0.96, *P* = 0.04) were less common in girls. A total of 329 (60%) had been in contact with specialist children's services, and this was more common in girls (OR 1.86, 95% CI 1.19–2.94, *P* = 0.007).

**Conclusions:**

There are several antecedents to suicide in young people, particularly girls, which are important in a multiagency approach to prevention incorporating education, social care, health services and the third sector. Some of these may also have contributed to the recent rise.

## Background

Suicide in young people is a major public health concern. Worldwide suicide is most common in young people and is the third leading cause of death for both girls and boys aged 15–19.^1^ In many countries, including the UK, suicide rates in children and young people have been rising.^2,3^ The 2018 data on suicide death registrations from the Office for National Statistics (ONS) show a 22% 1-year increase in suicide rate in under 25-year-olds, a greater rise than in any other age group.^4^ The UK suicide rate in girls aged under 20 is now the highest since recording began in 1981.^5^

Rates of self-harm are also rising. Self-harm rates in the UK, in both young people presenting to primary care services^6^ and within the general population,^7^ are increasing at a faster rate in girls than in boys.^6,7^ Self-harm is a strong risk factor for subsequent suicide,^6^ but many young people who self-harm are unknown to services.^8^ In those aged under 20, unlike other age groups, the rise in 2018 continues an increase that has been apparent since 2010.^3^ This rise appears to have followed a different pattern in boys and girls (see supplementary Fig. 1 available at https://doi.org/10.1192/bjo.2020.33). The rise in girls begins later (2013 *v.* 2010) and more than doubles by 2018. This increasing rate has coincided with concern over the mental health impact of social media^9,10^ and increasing demand for child and adolescent mental health services.^11,12^

## Aims

Previous research has highlighted multiple stressors that occur before suicide in children and young people.^13–18^ However, many studies are limited by small or specific samples (i.e. clinical samples or self-reports of suicidal behaviour) or take their information from register-based sources. We have reported initial 1-year findings from England from our national study of suicide in young people.^13^ In this paper we present full findings for all UK nations over 3 years – the largest population-based study of its kind to examine the personal narratives of those closest to the young person before they died. We have sought to understand the antecedents of suicide in young people that could have contributed to the recent rise and led to a different pattern in girls and boys. Our aim was to record contacts with services that could play a part in prevention. We also wanted to examine particular subgroups – ‘looked after children’, lesbian, gay, bisexual and transgender (LGBT) young people, and young people who had been bereaved.

In the UK, when a child or young person dies by suicide a range of investigations by official bodies can occur. Reports from these investigations are a rich source of information, providing detailed personal testimony from families, friends and professionals on the stresses the young person was facing. Using data collected from these investigations we aimed to: (a) report numbers and examine the antecedents of suicide by young people aged 10–19, including the characteristics of particular subgroups; (b) explore gender differences in these characteristics and (c) describe contacts with specialist services or agencies.

## Method

### Study setting

In this exploratory, national consecutive case series study we examined deaths by suicide (including probable suicide) in young people aged 10–19 in the UK between 1 January 2014 and 31 December 2016. We collected data from a range of investigations into their deaths by official bodies. We did not conduct new investigations.

### General population mortality data

National mortality data on young people who died by suicide were obtained from ONS (for deaths registered in England and Wales), National Records of Scotland (for deaths registered in Scotland) and the Northern Ireland Statistics and Research Agency (for deaths registered in Northern Ireland). Deaths classified as being the result of suicide or intentional self-harm (ICD-10 codes X60–X84) or events of undetermined intent (ICD-10 codes Y10–Y34, excluding Y33⋅9, Y87⋅0 and Y87⋅2) were included in the study, as is conventional in UK suicide research.^19^ Deaths receiving a narrative conclusion at coroner inquest were included if ONS procedures applied one of the ICD-10 codes listed above (this does not apply to deaths in Northern Ireland or Scotland). These deaths are collectively referred to as suicides.

### Data sources

#### Coroner inquest hearings/files or police death reports

Audio-recordings of inquest proceedings were requested for all suicide deaths from the senior coroner of the jurisdiction where the death occurred. Statements or depositions submitted as evidence during the inquest were requested where an audio-recording was unavailable. In Northern Ireland, where inquests are less likely to be held, witness statements and post-mortem reports were requested from the Northern Ireland Courts and Tribunal Service. For deaths in Scotland, redacted police death reports were requested from the Crown Office and Procurator Fiscal Service. We obtained information from coroner inquest hearings (or country equivalents) for 526 (88%) deaths. For 40 deaths the coroner (or equivalent) did not wish to or was unable to provide data, and in 29 data was not returned.

We examined the degree of agreement (concordance) between three researchers to ensure the interrater reliability of data extraction from a sample of 10% of coroner inquest hearings (*n* = 49) using Cohen's kappa analysis. Concordance rates for individual items were 52–100%. Where there were uncertainties or disagreement, the information was reassessed and concordance reached following discussion.

#### Child death investigations

In England, it is mandatory for Safeguarding Children Partnerships (SCPs) to review all child deaths up to the age of 18 via review processes conducted by a Child Death Overview Panel (CDOP). Anonymous Form C analysis proformas were requested from all SCPs in England where their respective CDOP had conducted a review into the death of a child by suicide or self-inflicted harm. Of 146 SCPs, 119 (82%) agreed to participate. Of these 119, 76 provided data, resulting in Form C returns on 118 (46%) people aged under 18. Thirty-three SCPs had not reviewed any deaths by suicide or self-inflicted harm in the study period, six were pending review and in four data were not returned. Twenty-seven (18%) SCPs did not participate.

#### Case reviews

Twenty case reviews (child safeguarding practice review in England, child practice review in Wales, case management review in Northern Ireland, and significant case review in Scotland; collectively referred to as case reviews in this paper) were obtained from the relevant SCP or from the National Society for the Prevention of Cruelty to Children national case review repository.

#### Criminal justice reports

For deaths that occurred between 1 January 2014 to 28 February 2015, the Prisons and Probation Ombudsman agreed to notify the study when any fatal investigation reports of deaths by apparent suicide in custody in people aged under 20 were published and available to download from their website. For deaths after 1 March 2015, identifiable details (i.e. name, age, and establishment where they died), are published by the Prisons and Probation Ombudsman, so additional notifications were not required to search for reports. In Northern Ireland, investigations into deaths in custody are published on the Prisoner Ombudsman for Northern Ireland website. Seven criminal justice reports were obtained.

#### National Confidential Inquiry into Suicide and Safety in Mental Health (NCISH) data

NCISH collects data on a UK-wide consecutive case series of people who die by suicide while under the recent care of mental health services. An explanation of NCISH data-collection methods has been previously published.^20^ Briefly, national data provide details on all people who die by suicide. Mental health providers then identify which of these had contact with mental health services in the 12 months before death. Clinical information is collected via a questionnaire completed by the senior professional responsible for the patient's care. Information from NCISH was obtained for 115 (19%) young people.

#### National Health Service (NHS) serious incident reports

If a suicide by a patient was identified from NCISH data, the medical director of the treating NHS trust or health board was asked to provide a copy of the serious incident report (or critical incident review, or serious adverse incident report, referred to as serious incident reports in this paper) describing the findings of an internal investigation of the patient's death. We obtained 97.

### Procedures

Information on antecedents of suicide were extracted from the data sources on to a proforma for aggregated analysis. Information was collected about demographic and family characteristics (relationship status, living circumstances), education (academic and exam pressures), medical history (physical health conditions), mental health history (diagnosis, self-harm), internet use, bullying, abuse, bereavement and service contact (mental healthcare, justice system and social care). Data items were determined *a priori* from the research literature and advice from people with specialist (lived) expertise. Antecedents were recorded if they were referred to in any of the data sources as having been present in the person's life at any time and specifically in the 3 months prior to their death (referred to as ‘recent’). Reference to a specific antecedent at an investigation implies that it was thought to be relevant to the death but not necessarily causal. Gender is used rather than sex throughout, denoting the individual's identity as reported by their family or friends during an official investigation of their death. Definitions have been previously published.^13^

### Statistical analysis

The denominator in all estimates was the total number of individuals on which at least one report was obtained (i.e. 544 individuals), unless otherwise specified. If an item (i.e. bereavement) was not recorded in any data source it was assumed to be absent or not relevant to the individual death. Pearson's chi-square test or Fishers exact test were used to examine associations between gender, particular subgroup (for example LGBT youth), and other characteristics. The estimated strength of the univariate association was measured by logistic regression models, adjusted for age, gender and presence of a diagnosis (previous research has shown the presence of any mental disorder to be associated with a higher risk of suicide^18,21^).

**Fig. 1:**
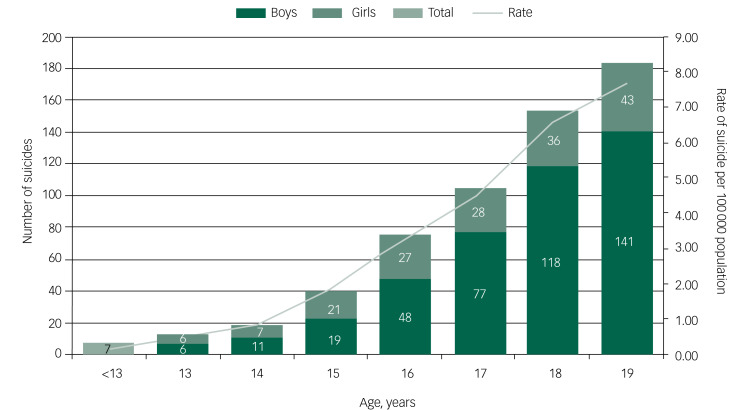
Number and rate of suicides, by age and gender.

Odds ratios (OR) and 95% CIs are presented. A Poisson regression model was used to compare the suicide rate (calculated using ONS mid-year population estimates, age 10–19, as denominators^22^) by age at death using the incident rate ratio (IRR). The reference age was 15 as it is the midpoint age for the sample and, in both genders, is the age at which the suicide rate notably increases (see Fig. 1). An IRR greater than 1.0 suggests an increased risk of suicide and 95% CIs were calculated for the precision of the IRRs. Stata version 15 was used for analysis and STROBE guidelines were followed (see supplementary data 1 for the STROBE checklist). We applied guidance from ONS on disclosure control to protect confidentiality, and supressed cell counts under three, including zero. Findings are combined for each UK nation.

### Ethics statement

The authors assert that all procedures contributing to this work comply with the ethical standards of the relevant national and institutional committees on human experimentation and with the Helsinki Declaration of 1975, as revised in 2008. All procedures involving human subjects/patients were approved by the National Research Ethics Service Committee North West (Greater Manchester South, UK; 15/NW/0184). Exemption under Section 251 of the NHS Act 2006, enabling access to confidential and identifiable information without informed consent in the interest of improving care, was obtained from the Health Research Authority Confidentiality Advisory Group (15/CAG/0120) and the Public Benefit and Privacy Panel for Health and Social Care (1617-0107).

## Results

**Fig. 2:**
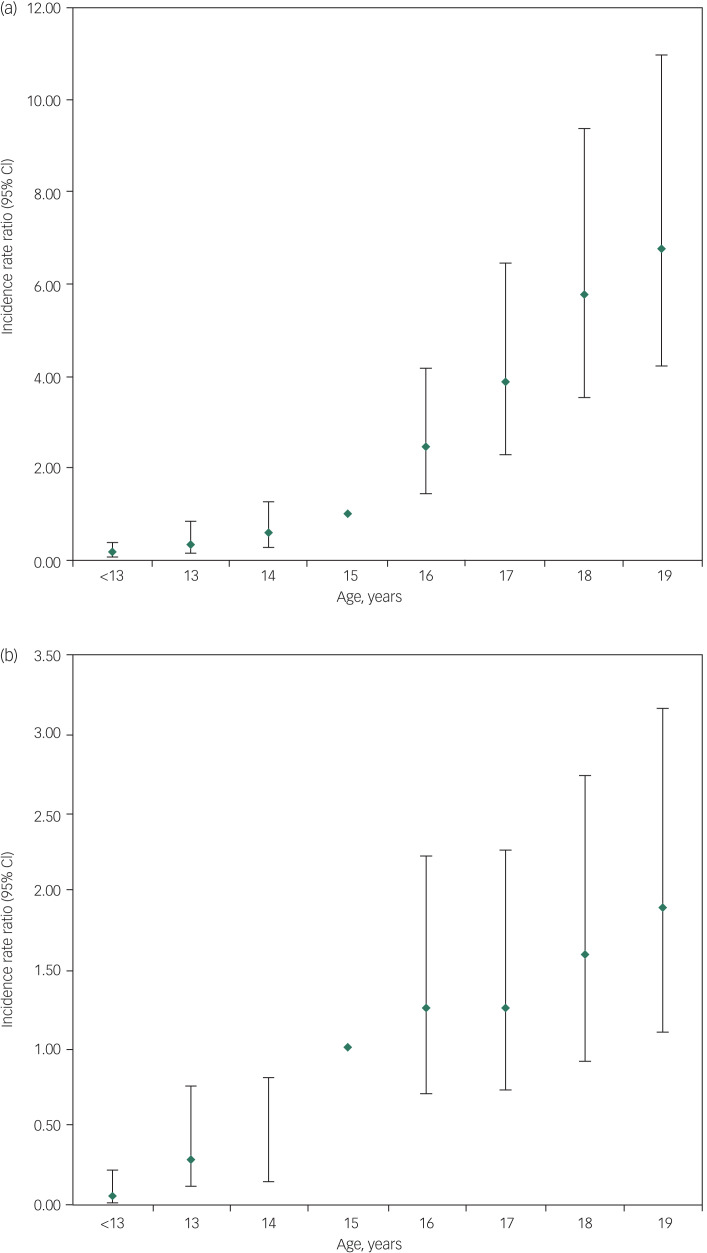
(a) Incidence of suicide in boys (aged under 20) (2014–2016). (b) Incidence of suicide in girls (aged under 20) (2014–2016).

Between 2014 and 2016, 595 people aged under 20 died by suicide in the UK, almost 200 deaths per year. Of these, 425 (71%) were male and 170 (29%) female. The number and rate of suicides increased with age, with 74% of deaths occurring in those aged 17–19 years (Fig. 1). Male suicide increased significantly with age (Fig. 2(a)), with the highest incidence at 18 (IRR = 5.78, 95% CI 3.56–9.39) and 19 (IRR = 6.77, 95% CI 4.19–10.93) compared with the reference age (age 15). In girls, the incidence of suicide was not significantly different from age 15 to 18 but increased significantly at age 19 (IRR = 1.88, 95% CI 1.11–3.16) compared with the reference age (age 15) (Fig. 2(b)).

The reference age is 15.

### Method of suicide

Hanging/strangulation was the most prevalent method (380, 64%) for both boys and girls (276, 65% (95% CI 60–69) *v.* 104, 61% (95% CI 53–69)), followed by multiple injuries (includes jumping from a height and railway deaths; 94, 16%) and self-poisoning (54, 9%). Other less frequent methods included gas inhalation (17, 3%), drowning (13, 2%) and firearms (6, 1%). Girls were more likely to die by self-poisoning than boys (28, 16% *v.* 26, 6%; OR = 3.03, 95% CI 1.72–5.34, *P* < 0.001). Opiates and opioids (such as tramadol, morphine) were the most commonly used substances taken in self-poisoning (21, 39% of all self-poisonings).

### Antecedents of suicide

Information was received from one or more data sources for 544 (91%), mainly from coroner inquest hearings (526, 88%). Table 1 shows the features of suicide by children and young people. The most common antecedents were self-harm, mental illness, academic pressures including exams or exam results, bereavement including by suicide, physical health conditions, drug or alcohol misuse and bullying (face-to-face and online). In 116 (21%) social isolation or recent social withdrawal had been reported. The most common physical health conditions may also have had a social impact – respiratory disease (62, 11%, especially asthma, 52, 10%) and dermatological problems (53, 10%, especially acne, 27, 5% and eczema, 22, 4%). The most common recent life events were about relationships, housing and the workplace.

**Table 1:** Demographic, social and clinical characteristics of children and young people who died by suicide, by gender (2014–2016)

Data items	Boys (*n* = 388)		Girls (*n* = 156)		Total (*n* = 544)		Univariate analyses					
							Unadjusted OR	95% CI	*P*	Adjusted OR^a^	95% CI	*P*
Sociodemographic												
Age, median (range)	18	(11–19)	17	(12–19)	18	(11–19)	–	–	–	–	–	–
Black, Asian and minority ethnic group, *n* (%)	28	(7)	19	(12)	47	(9)	1.78	0.96–3.30	0.07	1.82	0.96–3.42	0.07
LGBT and uncertain, *n* (%)	18	(5)	14	(9)	32	(6)	2.03	0.98–4.18	0.06	1.80	0.85–3.81	0.12
School pupil/student, *n* (%)	179	(46)	96	(62)	275	(51)	1.87	1.28–2.73	0.001	1.62	1.05–2.51	0.03
Employed (including apprenticeship), *n* (%)	94	(24)	21	(13)	115	(21)	0.49	0.29–0.82	0.006	0.63	0.36–1.09	0.10
Living alone, *n* (%)	19	(5)	8	(5)	27	(5)	1.05	0.45–2.45	0.91	1.13	0.47–2.72	0.79
Socially isolated (i.e. no or very few friends), *n* (%)	50	(13)	21	(13)	71	(13)	1.05	0.61–1.82	0.86	0.91	0.52–1.61	0.76
Family (parent/carer/sibling) factors, *n* (%)												
Mental illness	44	(11)	36	(23)	80	(15)	2.35	1.44–3.82	0.001	1.74	1.04–2.91	0.04
Physical illness	26	(7)	20	(13)	46	(8)	2.05	1.11–3.79	0.02	1.56	0.82–2.96	0.17
Substance misuse	27	(7)	17	(11)	44	(8)	1.64	0.86–3.09	0.13	1.20	0.62–2.34	0.59
Witnessing domestic violence	17	(4)	19	(12)	36	(7)	3.03	1.53–5.99	0.001	2.41	1.19–4.88	0.02
Abuse and neglect, *n* (%)												
Abuse (physical, emotional, sexual)	25	(6)	25	(16)	50	(9)	2.77	1.54–5.00	0.001	2.01	1.08–3.72	0.03
Neglect	14	(4)	9	(6)	23	(4)	1.64	0.69–3.86	0.26	1.25	0.51–3.04	0.62
Experience of bereavement, *n* (%)												
Bereaved	82	(21)	52	(33)	134	(25)	1.87	1.24–2.82	0.003	1.71	1.12–2.62	0.01
Bereaved by suicide	27	(7)	24	(15)	51	(9)	2.43	1.35–4.36	0.003	2.40	1.31–4.40	0.004
Bullying, *n* (%)												
Bullying (any)	56	(14)	46	(29)	102	(19)	2.48	1.59–3.87	<0.001	1.89	1.18–3.02	0.008
Face-to-face bullying	48	(12)	41	(26)	89	(16)	2.53	1.58–4.03	<0.001	1.93	1.18–3.14	0.009
Academic pressures, *n* (%)												
Academic pressures overall	106	(27)	68	(44)	174	(32)	2.06	1.40–3.03	<0.001	1.54^b^	1.00–2.38	0.05
Current or impending exams or exam results	41	(11)	37	(24)	78	(14)	2.63	1.61–4.30	<0.001	2.04^b^	1.20–3.49	0.009
Internet use, *n* (%)												
Suicide-related internet use (any)	78	(20)	50	(32)	128	(24)	1.87	1.23–2.85	0.003	1.52	0.98–2.36	0.06
Searching for information on suicide method	44	(11)	24	(15)	68	(13)	1.42	0.83–2.43	0.12	1.17	0.67–2.04	0.59
Posting suicidal ideas on social media	36	(9)	21	(13)	57	(10)	1.52	0.86–2.70	0.15	1.33	0.73–2.42	0.35
Online bullying	14	(4)	15	(10)	29	(5)	2.85	1.34–6.04	0.007	2.17	0.99–4.73	0.05
Visiting websites that may encourage suicide	11	(3)	5	(3)	16	(3)	1.13	0.39–3.32	0.82	0.97	0.32–2.91	0.95
Medical history, *n* (%)												
Physical health condition	105	(27)	59	(38)	164	(30)	1.64	1.11–2.43	0.01	1.56	1.04–2.34	0.03
Excessive alcohol use	85	(22)	32	(21)	117	(22)	0.92	0.58–1.45	0.72	0.89	0.55–1.43	0.62
Illicit drug use	153	(39)	43	(28)	196	(36)	0.58	0.39–0.88	0.01	0.54	0.35–0.83	0.006
Self-harm and suicidal ideas, *n* (%)												
Previous self-harm	159	(41)	108	(69)	267	(49)	3.24	2.18–4.91	<0.001	2.89	1.90–4.41	<0.001
Self-harm by cutting	66	(17)	53	(34)	119	(22)	2.51	1.64–3.84	<0.001	2.08	1.34–3.23	0.001
Self-harm by overdose	41	(11)	37	(24)	78	(14)	2.63	1.61–4.30	<0.001	2.54	1.51–4.25	<0.001
Serious recent episode of self-harm (requiring medical treatment)	47	(12)	47	(30)	94	(17)	3.13	1.98–4.95	<0.001	3.09	1.89–5.06	<0.001
Suicidal intent/ideas	217	(56)	107	(69)	324	(60)	1.72	1.16–2.55	0.007	1.38	0.91–2.10	0.13
Primary diagnosis, *n* (%)												
Any diagnosis of mental illness	136	(35)	81	(52)	217	(40)	2.00	1.37–2.92	<0.001	2.18	1.48–3.12	<0.001
Affective disorder (bipolar disorder and depression)	61	(16)	40	(26)	101	(19)	1.85	1.18–2.90	0.008	1.20	0.69–2.09	0.53
Anxiety/obsessive–compulsive/post-traumatic stress disorder	23	(6)	12	(8)	35	(6)	1.32	0.64–2.73	0.45	0.87	0.40–1.88	0.73
Recent events, *n* (%)												
Relationship breakup	75	(19)	41	(26)	116	(21)	1.49	0.96–2.30	0.07	1.47	0.94–2.30	0.09
Relationship problems	94	(24)	54	(35)	148	(27)	1.66	1.11–2.48	0.01	1.88	1.24–2.87	0.003
Housing problems	56	(14)	24	(15)	80	(15)	1.08	0.64–1.81	0.78	1.07	0.63–1.84	0.80
Workplace problems	68	(18)	15	(10)	83	(15)	0.50	0.28–0.91	0.02	0.52	0.28–0.96	0.04
Service contact (at any time), *n* (%)												
Any service contact	214	(55)	115	(74)	329	(60)	2.28	1.52–3.43	<0.001	1.86	1.19–2.94	0.007
Mental health services	151	(39)	90	(58)	241	(44)	2.14	1.47–3.12	<0.001	1.75	1.10–2.79	0.02
Social care or local authority services	50	(13)	49	(31)	99	(18)	3.10	1.97–4.85	<0.001	2.58	1.62–4.10	<0.001
Youth Offending Team or local police force	104	(27)	49	(31)	153	(28)	1.25	0.83–1.88	0.28	1.06	0.69–1.61	0.80
Looked after child, *n* (%)	21	(5)	21	(13)	42	(8)	2.72	1.44–5.14	0.002	2.51	1.30–4.83	0.006

LGBT, lesbian, gay, bisexual and transgender.

a. Adjusted by age and presence of a diagnosis.

b. Adjusted by age, gender, presence of a diagnosis, and being in education (i.e. were a school pupil/student).

Suicide-related internet use was reported for 128 (24%; Table 1). Of the 68 who had searched the internet for information on suicide method, 21 (4%) died by the method they had searched on – most often hanging/strangulation (*n* = 10). A total of 29 (5%) had been bullied online, 18 (3%) recently.

### Comparison of boys and girls

Table 1 presents a comparison of these antecedents in boys and girls, including after adjustment for age and mental illness. Many of the most frequent antecedents were more common in girls: witnessing domestic violence, abuse, bullying, academic pressures, bereavement, bereavement by suicide, physical health problems and mental illness. Self-harm was more often reported in girls, including recent serious self-harm requiring medical treatment. Girls were more likely to have had recent relationship problems, while boys more often had workplace problems. Illicit drug use but not alcohol misuse was more common in boys. Suicide-related internet use was more common in girls but in the adjusted analysis the difference was of only borderline significance.

#### LGBT groups

Twenty-eight (5%) were reported to have identified themselves as lesbian (*n* = 7), gay (*n* = 8), bisexual (*n* = 8) or transgender (*n* = 5) (LGBT) and four were uncertain of their sexual identity. Of this group (LGBT or uncertain), 20 (63%) were recorded as struggling with how they would tell family or friends or were experiencing internal turmoil about their sexual identity. Seventeen (53%) were aged under 18. Many antecedents of suicide in young people were more common in those identifying as LGBT, including abuse (5, 16%), bullying (9, 28%), previous self-harm (20, 63%) and suicidal ideas or intent (24, 75%; see supplementary Table 1). A significantly higher proportion of young people who identified as LGBT (or uncertain) had used the internet in ways that were suicide-related compared with those who did not identify as LGBT (14, 44% *v.* 128, 25%; OR 2.35, 95% CI 1.10–5.05, *P* = 0.03).

#### Looked after children

Forty-two (8%) had been looked after children at the time of death or previously. They had particularly high rates of substance misuse in the family and of being abused and/or neglected, and higher rates of housing problems (including having recently changed accommodation), social isolation, mental illness in the family, witnessing domestic violence, bereavement, excessive alcohol use and illicit drug use than young people not in care (see supplementary Table 1). Thirty-six (86%) had recent contact with at least one service, significantly more than the sample of young people as a whole (186, 37%; OR = 9.87, 95% CI 3.89–25.03, *P* < 0.001), but 20 (48%) were not in recent contact with mental healthcare.

#### Bereavement

There were 134 (25%) young people recorded as being bereaved by the death of a family member (105, 19%) or friend (35, 6%). Thirty-one (6%) had experienced multiple bereavements. Time since the bereavement varied (range 1–18 years). For most (61, 46%) the bereavement occurred more than 12 months earlier. In 67 (50%), the bereavement had occurred in the previous year, 23 (17%) in the 3 months prior to death. Bereavement added to existing adversities – many antecedents of suicide in young people were significantly more likely in those who had been bereaved than those who had not: disruption in the family environment through mental or physical illness and substance misuse, a history of neglect, excessive alcohol use, self-harm, and suicidal ideas (see supplementary Table 1). A total of 51 (9%) had been bereaved by suicide.

### Service contact

For 329 (60%) young people there had been contact with specialist services or agencies (excludes primary care or accident and emergency department) at some time; most often with mental health services (Table 1). Girls were more likely than boys to have had contact with all services except justice agencies, including in the 3 months before death (mental health services: 85, 22% *v.* 58, 37%; *P* < 0.001, social care services: 23, 6% *v.* 20, 13%; *P* < 0.001). It was more common for boys to have had no known contact with any services.

The ‘no contact’ group (215 young people, 40% of those who died), had low rates of mental and physical illness, self-harm and bereavement (Table 2). However, indications of risk were present in some who had no service contact, including 14% of those with a diagnosis of mental illness (from a general practice or at accident and emergency department), 22% who had previously self-harmed, and 38% who had expressed suicidal ideas and/or intent.

**Table 2:** Antecedents of suicide and ‘no contact’ or ‘multiple contact’ with front-line services^a^

	No contact (*n* = 215)					Multiple contact (*n* = 51)				
	*n* (%)	Unadjusted univariate analyses		Adjusted^a^ univariate analyses		*n* (%)	Unadjusted univariate analyses		Adjusted^a^ univariate analyses	
		OR (95% CI)	*P*	OR (95% CI)	*P*		OR (95% CI)	*P*	OR (95% CI)	*P*
Socially isolated	18 (8)	0.48 (0.27–0.84)	0.01	0.63 (0.33–1.17)	0.14	13 (25)	2.57 (1.29–5.10)	0.007	2.13 (1.03–4.40)	0.04
Family history										
Mental illness	15 (7)	0.30 (0.17–0.55)	0.001	0.41 (0.21–1.79)	0.008	25 (49)	7.66 (4.13–14.18)	<0.001	5.20 (2.70–10.03)	<0.001
Physical illness	7 (3)	0.25 (0.11–0.57)	0.001	0.32 (0.13–0.77)	0.01	9 (18)	2.64 (1.19–5.84)	0.02	1.66 (0.72–3.85)	0.23
Substance misuse	5 (2)	0.18 (0.07–0.46)	<0.001	0.19 (0.07–0.53)	0.001	15 (29)	6.67 (3.28–13.55)	<0.001	5.02 (2.35–10.72)	<0.001
Domestic violence	<3	—	—	—	—	15 (29)	9.37 (4.45–19.71)	<0.001	6.93 (3.10–15.54)	<0.001
Abuse	<3	—	—	—	—	25 (49)	18.00 (9.11–35.55)	<0.001	12.61 (6.13–25.96)	<0.001
Bereaved	30 (14)	0.35 (0.22–0.55)	<0.001	0.42 (0.26–0.69)	0.001	21 (41)	2.35 (1.30–4.27)	0.005	1.76 (0.94–3.31)	0.08
Bullying	16 (7)	0.23 (0.13–0.40)	<0.001	0.28 (0.15–0.53)	<0.001	16 (31)	2.16 (1.15–4.08)	0.02	1.21 (0.61–2.40)	0.58
Suicide-related internet use	36 (17)	0.52 (0.34–0.80)	0.003	0.60 (0.37–0.98)	0.04	18 (35)	1.90 (1.03–3.50)	0.04	1.29 (0.67–2.48)	0.44
Medical history										
Physical health condition	60 (28)	0.84 (0.57–1.22)	0.36	1.02 (0.67–1.57)	0.91	21 (41)	1.71 (0.95–3.09)	0.07	1.41 (0.76–2.62)	0.28
Excessive alcohol use	31 (14)	0.48 (0.30–0.75)	0.001	0.57 (0.34–0.94)	0.03	21 (41)	2.89 (1.59–5.28)	0.001	2.90 (1.51–5.57)	0.001
Illicit drug use	53 (25)	0.43 (0.29–0.62)	<0.001	0.47 (0.31–0.73)	0.001	28 (55)	2.36 (1.32–4.22)	0.004	2.72 (1.41–5.23)	0.003
Previous self-harm	48 (22)	0.14 (0.10–0.21)	<0.001	0.21 (0.14–0.33)	<0.001	43 (84)	6.45 (2.97–14.01)	<0.001	3.99 (1.74–9.13)	0.001
Suicidal intent/ideas	81 (38)	0.21 (0.15–0.31)	<0.001	0.30 (0.20–0.46)	<0.001	44 (86)	4.78 (2.11–10.83)	<0.001	2.97 (1.27–6.96)	0.01
Any diagnosis of mental illness	30 (14)	0.12 (0.08–0.19)	<0.001	0.13 (0.08–0.20)	<0.001	37 (73)	4.60 (2.42–8.73)	<0.001	4.50 (2.32–8.74)	<0.001
Looked after child	<3	—	—	—	—	23 (45)	20.49 (10.00–44.99)	<0.001	20.82 (9.16–47.33)	<0.001

a. Adjusted by age, gender and presence of a diagnosis.

Contact with multiple agencies (i.e. mental health and social care/local authority and justice agencies) was more likely in girls than boys. The ‘multiple contact’ group (51 young people, 9% of those who died) were more likely to have a family history of mental illness, substance misuse or domestic violence and higher rates of childhood abuse, social isolation, self-harm, alcohol and drug misuse (Table 2). Twenty-three (45%) of those in multiple contact were or had been looked after children.

## Discussion

### Main findings

The suicide rate in young people in the UK is currently rising.^3,23^ The recent lowering of the standard of proof threshold required for a suicide conclusion in England and Wales is expected to lead to a further increase in the number of deaths recorded as suicide in this age group.^24^ In this study, to our knowledge the first to investigate a complete national sample of individual suicides by young people, we sought to explore what stresses they face before they take their lives. In a 3-year period, we were notified of all suicides by people aged under 20 in the UK, almost 600 in total, the largest study of its kind. The suicide rate in this group increased every year from their mid- to late teens.

The most common suicide methods (hanging and multiple injuries) carry a high likelihood of fatality. A number of stresses were identified: family mental illness, childhood abuse or neglect, bullying, bereavement, academic stresses and physical health conditions. Other antecedents included mental illness, self-harm and illicit drug or alcohol misuse. Suicide-related internet use was reported in a quarter of the young people, including a significant minority who had communicated suicidal ideas on social media, although the commonest type of internet use was searching for information on suicide methods.

Although the majority of suicides in this age group are in boys, there have been particular concerns about rising rates of suicide and self-harm in girls and young women. Many of the stresses we identified were significantly more common in girls. These included childhood-related antecedents’ such as family mental illness and domestic violence, abuse, parental bereavement, bullying, current or impending exams or exam results, physical health conditions and self-harm. The only experiences more common in boys were illicit drug use and workplace problems. Self-poisoning was a more common suicide method in girls, in line with what is known about gender differences in method lethality,^3^ with opioids the drug type most often taken, adding to concerns about their availability.^25^ There have been few recent studies examining gender difference in antecedents of suicide. We found girls were more likely than boys to have contact with services, including in the 3 months before death, consistent with previous studies examining coroner records.^26^

### Strengths and limitations

Information for the study came mainly from coroners, who independently take evidence from several sources, including the personal narrative of families, friends and professionals in contact with the young person prior to their death. However, several limitations arise from our use of these sources. First, there may be the potential for subjective bias in extracting information from coroner inquests. Second, information may be subject to recall bias, potential gender differences in disclosure, and variations in completeness; information on suicide-related internet use, for example, may be underestimated in deaths where, during the investigation for the coroner, the police were unable to access the young person's electronic media, and for deaths in Scotland, police reports were generally less detailed than coroner data.

Third, some figures may be overestimates as families and others ‘search for meaning’ after a suicide emphasising factors they see as most relevant, whereas other factors, particularly in sensitive areas (such as sexuality, abuse), may be underreported. We also acknowledge the antecedents we identified may not be comprehensive. Fourth, this was an observational, not a risk factor, study and we did not use a control group. Obtaining equivalent sources of data on suitable non-suicide controls is difficult^27^ and a controlled study would have been difficult to achieve, in part because of the ethical implications in contacting families. Previous psychological autopsy studies in which families and others have been interviewed along with control families have raised doubts about equivalence – the fact of suicide itself, its impact on disclosure, and the reluctance of potential controls distort any comparison.^28^ The findings we identify in our study, although they cannot be linked causally to suicide, do describe the adversities young people were facing prior to death – they were taken from personal narratives, which were discussed at inquest for the reason that the informant or coroner felt they were relevant to the person's death.

Fifth, the data we used were not designed for research purposes and content detail varied. Sixth, the findings are presented as UK-wide aggregate figures and are driven by the larger number of suicides in England.

### Interpretation of findings

The range of antecedents identified in this study highlights the need for a broadly based approach to suicide prevention with many agencies contributing: support for families through social care, antibullying policies in schools and the workplace, safer prescribing of opioids in primary care, third-sector support for bereaved families, mental health services offering urgent access, psychosocial assessment after self-harm and better understanding of how to look after emotional health through schools, universities, public health and the media. Some groups have specific needs: housing and mental health support for looked after children, antibullying measures for LGBT groups, although for them a more fundamental route to prevention lies in social attitudes towards diversity. Internet companies have a role in improving online safety, not just through social media but in reducing the accessibility of information on suicide methods.

From this study, we cannot say whether these experiences have contributed to the rise in suicide in young people over the past decade. Exposure to internet risks has presumably increased, and possibly academic stresses, and the use of more dangerous suicide methods. In particular, self-harm rates have risen, fuelled by a growing perception of self-harm as a way of coping with stress.^7^ Social learning through media exposures may also have a role in increasing suicide rates particularly for young people who are more vulnerable to contagion.^29^ Suicide among young people may also be driven by society-wide factors beyond the scope of our data collection on individuals: economic adversity in a period of austerity, demand for mental healthcare that services have struggled to respond to, insecurity in jobs and housing, and fears about opportunity and the environment. These are areas for future investigation.

## Data Availability

All authors had full access to all the data in the study and take responsibility for the integrity of the data and the accuracy of data analysis; access to the data is currently ongoing. Data from this study cannot be shared because of information governance restrictions in place to protect confidentiality. Access to data can be requested via application to the Healthcare Quality Improvement Partnership (www.hqip.org.uk/national-programmes/accessing-ncapop-data/).

## Supplementary material

For supplementary material accompanying this paper visit http://dx.doi.org/10.1192/bjo.2020.33. click here to view supplementary material
