# Supplementary material for: Children and young people who die by suicide: childhood-related antecedents, gender differences and service contact
Source: BJPsych Open. 2020 May 11;6(3):e49. doi: 10.1192/bjo.2020.33 (PMC7331086; doi:10.1192/bjo.2020.33)
Supplement: Supplementary file 1 [file S2056472420000332sup001.zip › [Rodway] Online-only supplement Table 1 Sub-group data_clean.docx]

**Supplementary Table 1** *Antecedents of suicide in particular sub-groups*

| **Data item** | **LGBT groups (Number=32)** | | **Looked after children (Number=42)** | | **Bereaved young people (Number=134)** | |
| --- | --- | --- | --- | --- | --- | --- |
|  | **Adjusted* OR (95% CI)** | ***P* value** | **Adjusted* OR (95% CI)** | ***P* value** | **Adjusted* OR (95% CI)** | ***P* value** |
| **Socio-demographic**  Black, Asian and minority ethnic group | 0.29 (0.34-2.23) | 0.24 | 0.96 (0.32-2.90) | 0.95 | 0.45 (0.20-1.05) | 0.07 |
| LGBT and uncertain | --- | --- | 1.46 (0.47-4.53) | 0.51 | 0.58 (0.23-1.48) | 0.26 |
| School pupil/student | 1.45 (0.63-3.33) | 0.38 | 0.33 (0.15-0.72) | 0.005 | 0.56 (0.35-0.88) | 0.01 |
| Employed (including apprenticeship) | 0.84 (0.30-2.37) | 0.74 | 0.99 (0.42-2.33) | 0.99 | 1.98 (1.20-3.28) | 0.007 |
| Living alone | 2.38 (0.64-8.85) | 0.19 | 5.55 (2.18-15.16) | <.001 | 1.52 (0.65-3.56) | 0.34 |
| Socially isolated | 1.18 (0.43-3.22) | 0.75 | 2.37 (1.10-5.09) | 0.03 | 1.52 (0.87-2.63) | 0.14 |
| **Family history**  Mental illness | 0.53 (0.17-1.68) | 0.28 | 3.77 (1.82-7.82) | <.001 | 2.44 (1.44-4.13) | 0.001 |
| Physical illness | 0.23 (0.03-1.77) | 0.16 | 2.41 (1.00-5.81) | 0.05 | 4.88 (2.55-9.37) | <.001 |
| Substance misuse | 0.91 (0.25-3.28) | 0.89 | 15.99 (7.16-35.70) | <.001 | 3.01 (1.56-5.82) | 0.001 |
| Witnessing domestic violence | 0.28 (0.04-2.23) | 0.23 | 5.05 (2.12-12.02) | <.001 | 2.32 (1.13-4.77) | 0.02 |
| **Abuse and neglect**  Abuse (physical, emotional, sexual) | 1.31 (0.44-3.87) | 0.62 | 17.72 (7.98-39.34) | <.001 | 1.86 (0.98-3.53) | 0.06 |
| Neglect | 0.55 (0.07-4.35) | 0.57 | 24.18 (9.07-64.48) | <.001 | 4.83 (1.99-11.72) | 0.001 |
| **Experience of bereavement**  Bereaved | 0.57 (0.55-3.14) | 0.24 | 2.27 (1.17-4.41) | 0.02 | --- | --- |
| Bereaved by suicide | 0.52 (0.11-2.29) | 0.39 | 1.26 (0.49-3.27) | 0.63 | --- | --- |
| **Bullying**  Bullying (any) | 1.31 (0.55-3.14) | 0.54 | 1.03 (0.46-2.28) | 0.95 | 1.63 (0.99-2.67) | 0.05 |
| Face-to-face bullying | 1.60 (0.67-3.85) | 0.29 | 0.73 (0.30-1.80) | 0.50 | 1.45 (0.86-2.44) | 0.17 |
| **Academic pressures**  Academic pressures overall | 1.10 (0.49-2.47)^a^ | 0.81 | 0.61 (0.26-1.44)^a^ | 0.26 | 0.74 (0.45-1.21)^a^ | 0.23 |
| Current or impending exams or exam results | 0.82 (0.28-2.36)^a^ | 0.71 | 0.34 (0.07-1.58)^a^ | 0.17 | 0.55 (0.27-1.10)^a^ | 0.09 |
| **Internet use**  Suicide-related internet use (any) | 2.35 (1.10-5.05) | 0.03 | 1.48 (0.73-3.01) | 0.27 | 1.85 (1.18-2.91) | 0.008 |
| Searching for information on suicide method | 2.31 (0.97-5.51) | 0.06 | 0.45 (0.13-1.53) | 0.20 | 1.05 (0.58-1.90) | 0.88 |
| Posting suicidal ideas on social media | 5.13 (2.27-11.60) | <.001 | 2.71 (1.18-6.24) | 0.02 | 2.21 (1.21-4.01) | 0.01 |
| Online bullying | 0.35 (0.04-2.83) | 0.33 | 2.09 (0.72-6.10) | 0.18 | 1.36 (0.60-3.11) | 0.46 |
| Visiting websites that may encourage suicide | 5.70 (1.68-19.32) | 0.005 | 0.73 (0.09-5.86) | 0.77 | 0.96 (0.30-3.08) | 0.94 |

**Supplementary Table 1 (continued)** *Antecedents of suicide in particular sub-groups*

| **Data items** | **LGBT groups (Number=32)** | | **Looked after children (Number=42)** | | **Bereaved young people (Number=134)** | |
| --- | --- | --- | --- | --- | --- | --- |
|  | **Adjusted* OR (95% CI)** | ***P* value** | **Adjusted* OR (95% CI)** | ***P* value** | **Adjusted* OR (95% CI)** | ***P* value** |
| **Medical history**  Physical health condition | 1.11 (0.51-2.38) | 0.80 | 0.50 (0.23-1.09) | 0.08 | 1.12 (0.73-1.71) | 0.61 |
| Excessive alcohol use | 0.66 (0.24-1.78) | 0.41 | 2.26 (1.13-4.49) | 0.02 | 2.04 (1.29-3.22) | 0.002 |
| Illicit drug use | 1.19 (0.55-2.56) | 0.67 | 2.38 (1.21-4.67) | 0.01 | 0.83 (0.54-1.28) | 0.40 |
| **Self-harm and suicidal ideas**  Previous self-harm | 1.49 (0.66-3.40) | 0.34 | 2.02 (0.94-4.33) | 0.07 | 1.98 (1.28-3.07) | 0.002 |
| Self-harm by cutting | 1.84 (0.83-4.06) | 0.13 | 1.86 (0.93-3.72) | 0.08 | 1.26 (0.78-2.01) | 0.34 |
| Self-harm by overdose | 0.45 (0.13-1.59) | 0.21 | 0.88 (0.39-2.10) | 0.78 | 1.76 (1.03-2.99) | 0.04 |
| Serious recent episode of self-harm (requiring medical treatment) | 1.06 (0.41-2.75) | 0.91 | 1.63 (0.76-3.51) | 0.21 | 2.44 (1.48-4.02) | <.001 |
| Suicidal intent/ideas | 1.89 (0.80-4.47) | 0.15 | 1.32 (0.63-2.75) | 0.46 | 2.18 (1.38-3.45) | 0.001 |
| **Primary diagnosis**  Any diagnosis of mental illness | 1.46 (0.70-3.03) | 0.31 | 2.04 (1.06-3.94) | 0.03 | 1.81 (1.21-2.71) | 0.004 |
| Affective disorder (bipolar disorder and depression) | 1.16 (0.48-2.80) | 0.74 | 0.72 (0.31-1.71) | 0.46 | 1.35 (0.83-2.19) | 0.22 |
| Anxiety/Obsessive compulsive/Post-traumatic stress disorder | 0.96 (0.22-4.21) | 0.95 | 0.64 (0.15-2.81) | 0.56 | 1.84 (0.89-3.79) | 0.10 |
| **Recent events**  Relationship break-up | 0.96 (0.40-2.30) | 0.93 | 1.56 (0.77-3.15) | 0.22 | 1.50 (0.95-2.38) | 0.08 |
| Relationship problems | 1.65 (0.78-3.52) | 0.19 | 1.27 (0.64-2.52) | 0.50 | 1.05 (0.67-1.64) | 0.84 |
| Housing problems | 1.08 (0.39-2.93) | 0.89 | 5.77 (2.89-11.52) | <.001 | 1.70 (1.01-2.87) | 0.05 |
| Workplace problems | 0.65 (0.19-2.26) | 0.50 | 0.89 (0.35-2.29) | 0.81 | 0.95 (0.54-1.69) | 0.86 |
| **Service contact (at any time)**  Mental health services | 1.14 (0.46-2.83) | 0.78 | 2.82 (1.22-6.53) | 0.02 | 2.21 (1.36-3.61) | 0.001 |
| Social care or local authority services | 1.12 (0.46-2.74) | 0.80 | --- | --- | 2.00 (1.23-3.24) | 0.005 |
| Youth Offending Team or local police force | 1.04 (0.47-2.30) | 0.92 | 6.37 (3.16-12.82) | <.001 | 1.76 (1.15-2.69) | 0.009 |
| Child in care | 1.42 (0.46-4.43) | 0.54 | --- | --- | 2.29 (1.19-4.44) | 0.01 |
| Multi-agency contact | 0.88 (0.27-2.83) | 0.83 | 20.40 (9.15-46.46) | <.001 | 1.81 (0.96-3.40) | 0.07 |
| No service contact | 0.82 (0.63-1.97) | 0.66 | --- | --- | 0.43 (0.26-0.70) | 0.001 |

***** Adjusted by age, gender and presence of a diagnosis. ^a^ Adjusted by age, gender, presence of a diagnosis, and being in education (i.e. were a school pupil/student)

* Boys and girls have been combined because of low numbers.
