# Supplementary figures and images for: Children and young people who die by suicide: childhood-related antecedents, gender differences and service contact
Source: BJPsych Open. 2020 May 11;6(3):e49. doi: 10.1192/bjo.2020.33 (PMC7331086; doi:10.1192/bjo.2020.33)

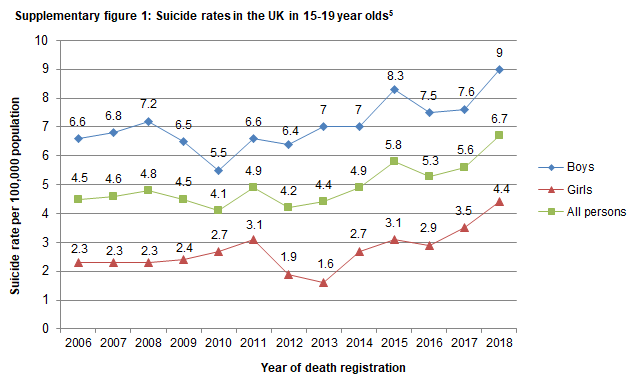

Supplement: Supplementary file 1 [file S2056472420000332sup001.zip › [Rodway] Online supplement only_Fig 1. Suicide rates in the UK in 15-19 year olds.tif]
